# The Role of the Tumor Microenvironment in Developing Successful Therapeutic and Secondary Prophylactic Breast Cancer Vaccines

**DOI:** 10.3390/vaccines8030529

**Published:** 2020-09-14

**Authors:** Benjamin Gordon, Vijayakrishna K. Gadi

**Affiliations:** 1 Department of Physiology and Biophysics, University of Illinois College of Medicine, Chicago, IL 60612, USA; 2 Medical Scientist Training Program, University of Illinois College of Medicine, Chicago, IL 60612, USA; 3 Division of Hematology and Oncology, University of Illinois Cancer Center, University of Illinois at Chicago, Chicago, IL 60612, USA; vkgadi@uic.edu

**Keywords:** breast cancer, cancer vaccines, tumor microenvironment, checkpoint inhibitors

## Abstract

Breast cancer affects roughly one in eight women over their lifetime and is a leading cause of cancer-related death in women. While outcomes have improved in recent years, prognosis remains poor for patients who present with either disseminated disease or aggressive molecular subtypes. Cancer immunotherapy has revolutionized the treatment of several cancers, with therapeutic vaccines aiming to direct the cytotoxic immune program against tumor cells showing particular promise. However, these results have yet to translate to breast cancer, which remains largely refractory from such approaches. Recent evidence suggests that the breast tumor microenvironment (TME) is an important and long understudied barrier to the efficacy of therapeutic vaccines. Through an improved understanding of the complex and biologically diverse breast TME, it may be possible to advance new combination strategies to render breast carcinomas sensitive to the effects of therapeutic vaccines. Here, we discuss past and present efforts to advance therapeutic vaccines in the treatment of breast cancer, the molecular mechanisms through which the TME contributes to the failure of such approaches, as well as the potential means through which these can be overcome.

## 1. Introduction

Immunotherapy has revolutionized the treatment paradigm for several cancers. While such approaches show promise in many solid tumors [1,2,3,4,5,6,7], progress for immunotherapy in breast cancer has been difficult [8]. This is particularly true for therapeutic vaccines, which have yet to show significant clinical efficacy in breast cancer [9]. While several barriers to efficacy have been suggested, there is a growing body of evidence supporting the tumor microenvironment (TME) as an important consideration in breast cancer immunotherapy [10].

**Figure 1:**
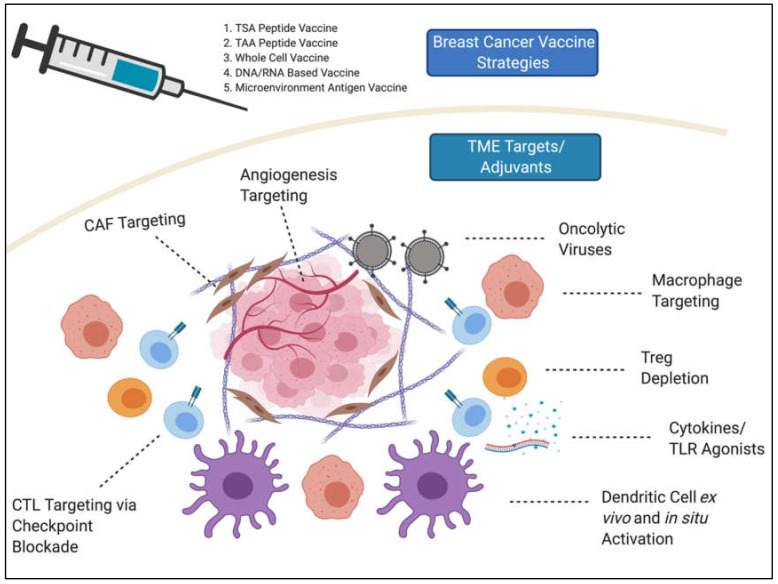
Awakening the tumor microenvironment as a means to increase efficacy of therapeutic breast cancer vaccines. Peptide, nucleotide, and whole cell-based vaccines have been tested for many years but have failed in the clinic partly because of an immunosuppressive tumor microenvironment (TME). Increased understanding of the TME and novel therapeutics to increase anti-tumor immune properties have increased vaccine promise in pre-clinical models. This paradigm is currently being tested in clinical trials. TME, tumor microenvironment; TSA, tumor specific antigen; TAA, tumor associated antigen; CAF, cancer associated fibroblast; Treg, regulatory T-cells; CTL, cytotoxic T lymphocytes; TLR, toll-like receptor. (Figure created with BioRender).

The TME has several roles in breast cancer etiology [11,12], and comprises heterogeneous populations of immune cells, fibroblasts, vascular endothelial cells, and extracellular matrix at both primary and metastatic sites [11]. Accordingly, the breast TME is often highly immunosuppressive, contributing to the relative failure of several immunotherapies [13,14]. Here, we discuss attempts to advance therapeutic vaccines in breast cancer, as well as factors within the breast TME that serve as barriers to their therapeutic efficacy and the potential means through which they can be overcome (Figure 1).

## 2. Breast Cancer Vaccines and the Microenvironment Hurdle

The goal of breast cancer vaccines is to induce a robust specific immune attack against antigens related to a patient’s tumor [15]. Tumor specific antigens (TSA) are antigens restricted to the tumor, such as neoantigens, cancer-testis antigens, and tumor-virus antigens, which have been discussed elsewhere [16]. New sequencing, quality control analysis, and bioinformatic pipelines have now made it possible to predict patient overall survival (OS) based on neoantigen binding affinity to MHC I and MHC II molecules [17]. This understanding opens the opportunity for personalized approaches to gauge immunogenic potential in individual patients. Tumor associated antigens (TAA), as opposed to TSAs, can be found in normal tissue, but are overexpressed in the tumor. For breast cancer, common TAAs include human epidermal growth factor-2/neu (HER2/neu), mucin-1 (MUC1), p53, carcinoembryonic antigens (CEA), and human telomerase reverse transcriptase (hTERT) [16]. Both TSAs and TAAs have been evaluated for suitability towards vaccine development in breast cancer in preclinical and early-clinical studies in order to elicit a CD8+ T-cell (cytotoxic T lymphocyte, or CTL) and Natural Killer Cell (NKT) response. 

DNA, RNA, and whole cell vaccines are other emerging therapeutic strategies against breast cancer, which are summarized in Table 1. In addition to the antigen presentation from vaccines, damage associated molecular patterns (DAMPS), which are certain nucleic acids or proteins emitted from dying cells, can bind toll like receptors (TLRs) of antigen presenting cells (APCs) of the innate immune system to trigger Type I interferon (IFN) responses [13]. DAMPs can be augmented by radiation therapy or other cytotoxic therapies, as will be discussed more below.

Most breast cancer (BC) vaccine development has focused on therapeutic use and/or secondary prophylaxis. While breast cancer vaccines show promise in preclinical experiments and appear to be safe to human patients [18], clinical responses are usually limited. In addition to tumor-intrinsic resistance mechanisms, the cancer microenvironment is a major reason for the frustrating clinical results indicating that vaccines have yet to help patients in a meaningful way [14].

Whereas antibody titers are major determinants for prophylactic pathogen vaccines, breast cancer vaccines aim to induce a sterile cytotoxic cell mediated effect against the tumor. Even with proper antigen presentation, however, the microenvironment can hinder proper vaccine mediated CD8+ T-cell responses in multiple ways. First, there is a physical barrier presented by the microenvironment. The stroma and extracellular matrix present a physical obstacle to T-cell infiltration [19,20]. Moreover, pathological tumor vasculature may hinder proper leukocyte infiltration into the tumor, as adhesion molecules and chemokines present in physiological inflammation may not be available [21]. In addition to physical barriers, the cellular makeup of the TME can be immunosuppressive, with an immune-inhibiting subset of T-cells, macrophages, dendritic, and myeloid derived suppressor cells (MDSCs) blunting an attack [11]. During effective responses to microbes, the innate and adaptive immune systems synergize for a proper immune attack. However, in the TME, there is an anti-inflammatory, pro-angiogenic, matrix-deposition, and tissue remodeling state in which the adaptive and innate cells of the BC niche are not able to promote beneficial immune responses. From an evolutionary point of view, this type of regulation in sterile immunity prevents autoimmune disease. However, for BC vaccines, an inflammatory TME is needed for sterile immunity against breast cancer cells. There are currently preclinical studies and clinical trials ongoing to circumvent this problem in two significant ways:

1. In situ TME modulation strategy: An emerging strategy to potentiate the effects of breast cancer vaccines is to use a priming agent to reprogram a highly immunosuppressive TME to take on a more pro-inflammatory state, thereby enhancing the presentation of tumor antigen. This process aims to take advantage of the physiologic response to microbial infections, in which pathogen-associated molecular patterns (PAMPs) are recognized by innate immune cells via by toll-like receptors (TLRs) and other pathogen sensing receptors. Parallel to the innate immune response, these invading microbes are also endocytosed, processed, and presented by APCs, allowing for interface between innate and adaptive immune systems. Antigen is presented on either MHC class I or MHC class II molecules, allowing for recognition by CD8+ or CD4+ T-cells, respectively. This is the first step in effector T-cell activation, which also requires additional co-stimulatory signals including association between the CD80 ligand on APCs and the CD28 receptor on T-cells. Cancer vaccines rely on a similar series of events to activate T-cell immunity but lack the infectious etiology to effectively initiate the above cascade. Hence, in the absence of associated strategies to foster antigen presentation to tumor-reactive T-cells within the TME, the effects of therapeutic vaccines may be limited. Several strategies to address this have been explored in the setting of therapeutic vaccines, including depletion of immunosuppressive cell subsets such as Tregs, additional stimulation of professional APCs, suppression of T-cell “stop signals” (i.e., PD-1/PD-L1, CTLA-4) via checkpoint inhibitors, and a molecular “mimic” of a microbial infection using either TLR agonists or oncolytic viruses. Recent progress for such approaches is discussed in detail below.

2. TME antigen targeting strategy: An additional strategy for breast vaccination is to use antigens derived from the surrounding tumor microenvironment rather than from tumor cells themselves. One potential advantage of this strategy is that resident-cells in the TME are likely more genomically stable than breast cancer cells themselves, which often display deficits in DNA repair. This genomic stability may prevent further immunoediting, and the eventual evasion from CTL attack through changes in the antigen repertoire. Several potential targets for therapeutic vaccines have been proposed, namely those related to the tumor vasculature and stromal cells, as summarized below.

## 3. T-Cells

Tumor infiltrating lymphocytes (TILs) are a significant predictor of prognosis in patients with breast cancer, especially for those with triple negative breast cancer (TNBC, ER-PR- without HER2 amplification) or certain ER+ subtypes [22,23,24]. However, infiltration of the tumor by CD8+ T-cells is insufficient by itself to immunologically clear the tumor. T-cells in the tumor microenvironment undergo exhaustion, senescence, and/or anergy, as CD8+ T-cells are not receiving proper co-stimulation or undergo chronic stress and ageing. Studies of CD8+ T-cell subset analysis highlights the heterogeneity of CTLs and informs on their various functions. For instance, CD39+ CD8+ T-cells are exhausted CTLs in BC that have compromised IL-2 and TNFα production, which foster primary and metastatic tumor growth [25]. CD39 is an ATP ectonucleotidase, which hydrolyzes ATP and suppresses an ATP-driven pro-inflammatory state. Moreover, using single-cell (sc) RNA-seq to profile CTLs within the breast cancer microenvironment, a large proportion express an exhausted phenotype [26,27]. The proper identification of effector CD8+ T-cells, as opposed to pure quantification of CD8+ TILs, may be instrumental in identifying targets for immunotherapy. Savas et al., for instance, used scRNA-seq to identify tissue-resident memory CD103+ CD8+ T-cells that are associated with improved prognosis and increased immunosurveillance [26].

BC vaccines are a poor therapeutic without their main ally: effective and healthy T-cells. Checkpoint signaling, such as through PD-1/PD-L1, is a key characteristic of CD8+ T-cell exhaustion. Moreover, infiltration of suppressive immune cells into the TME can further contribute to an underwhelming anti-tumor response. Therefore, two strategies that have been employed to get around these hurdles is (1) checkpoint blocking antibodies as a BC vaccine adjuvant and (2) strategies that create a pro-inflammatory TME by regulatory T-cell (Treg) depletion.

### 3.1. Checkpoint Blockades as Adjuvants to BC Vaccines

As discussed, a highly immunosuppressive TME impedes the effector function of tumor infiltrating lymphocytes. Thus, the T-cells residing in the breast TME are frequently anergic or functionally exhausted [25,28]. Beyond functioning as surrogate markers of T-cell exhaustion, immune checkpoints such as PD-1/PD-L1 have important roles in maintaining the immunosuppressive niche in the TME, and there is emerging evidence suggesting that some BC patients may derive clinical benefit from immune checkpoint inhibitors. For instance, the combination of Atezolizumab (Tecentriq) and nab-paclitaxel has now been approved for PD-L1+ TNBC [29]. While tumor PD-L1 positivity by immunohistochemistry has long been considered the gold standard for predicting responses to PD-1/PD-L1 inhibition, there is now mounting evidence that PD-1/PD-L1 expression on tumor infiltrating lymphocytes may also have predictive value, though this requires further investigation [30,31].

Beyond the expression of PD-1/PD-L1, there are several other clinically useful predictors of responses to immune checkpoint inhibition, most notably a high microsatellite instability phenotype (MSI-H) and/or an increased tumor mutation burden (TMB). Mismatch repair deficiency has been shown to strongly predict responses to immune checkpoint inhibition in a variety of solid tumor types [32]. In cases of high MSI-H and TMB, the increased mutational burden leads to a corresponding increase in associated neoantigens, which is a potentially significant consideration when predicting checkpoint blockade response. While MSI-H tumors have shown favorable responses to immune checkpoint inhibition in pan cancer trials [33], it is important to note that MSI-H is uncommon in BC, and may have limited utility in stratifying BC patients for therapy [34]. Although TMB is less established as a predictor of drug responses in BC as in other cancers [35], there is emerging evidence that TMB has significant impact in directing the local immune responses in TNBC [36]. While TMB scoring is still not well established in BC clinical practice, an intermediate or high TMB score across all BC subtypes was associated with an increase in tumor infiltrating CTLs and, rather interestingly, DNA damage repair gene mutations (*BRCA1/2*) [37]. This is an ongoing area of research, and murine models of TMB-high TNBC are showing early promise in substantiating TMB status as clinically useful predictor of checkpoint blockade response [38].

Recent preclinical and clinical progress testing immune checkpoint inhibitors in BC has compelled researchers and clinicians to combine these approaches with therapeutic vaccines. There are several ongoing clinical trials using anti-PD-1 pembrolizumab (Keytruda) [39], anti-PD-L1 avelumab (Bavencio), anti-PD-L1/TGF-β trap fusion M7824, anti-CTLA-4 tremelimumab, and anti-PD-1 durvalamab (Imfinzi) as adjuvants to various BC vaccines (see Table 2). In the clinical setting, for instance, a p53-expressing viral-based vaccine with pembrolizumab adjuvant identified a marked increase in p53-specific CD8+ effector T-cells with clinical response in two patients, one of whom had TNBC [39].

Functioning as vaccine adjuvants, drugs targeting checkpoint molecules have shown remarkable promise in the preclinical setting [40,41,42]. In one study, a soluble PD-1-based TWIST1 DNA vaccine in conjunction with anti-CTLA-4 antibody significantly reduced tumor growth and lung metastasis, presumably by increasing IFN-γ+ TNFα+ CD8+ T-cells [43]. Many other vaccine checkpoint blocking adjuvant combinations similarly increased CD8+ T and NK cell effector infiltration while causing tumor regression using different BC vaccine and adjuvant strategies [40,41,44,45,46,47]. A 4T1 model using autologous whole cell vaccine and anti-PD-1 antibodies not only increased CD8+ T-cell infiltration, but also reduced immunosuppressant CD68+ tumor associated macrophages (TAMs) and Gr-1+ CD11b+ myeloid derived suppressor cells (MDSCs) [42]. TAMs can be M1 (pro-inflammatory) or M2 (pro-tumorigenic) polarized; anti-PD-1 significantly reduced M2 polarized TAMs while favoring proinflammatory state as shown by increased IFN-γ+ CD68+ cells. In yet another demonstration of how checkpoint blockade can lead to a pro-inflammatory TME, Hassannia and colleagues downregulated PD-1 and PD-L1 via siRNA-based nanoparticles and used a dendritic-based BC vaccine to eliminate Treg cells in the TME [46]. Therefore, not only do checkpoint adjuvants stimulate T effector cells, but they regulate immunosuppressive cells in the TME.

Interestingly, many of the BC vaccine/checkpoint blockade combinations also significantly affected CD4+ helper T-cell contribution in the TME. Similar to TAM polarization, CD4+ helper T-cells can be Th1 (proinflammatory) or Th2 (pro-tumorigenic) polarized in the TME. When CD4+ T-cells were specifically deleted in a murine HER2+ BC model using a HER2-based dendritic vaccine in combination with anti-PD-1 and anti-PD-L1, the efficacy of the therapy was compromised [47]. Without CD4+ T-cell abrogation, the researchers showed that there was a strong IFN-γ based Th1 polarized response in the combination therapy group. This strategy led to a remarkable 400% survival rate in treated group compared to control mice. Of note, the CD4/CD8 T-cell response was dependent on MHC class used in the vaccine production, as MHC I based vaccine duly restricted the immune response to CD8+ T-cells. A high CD4/CD8 ratio is associated with worse patient prognosis in BC settings [48]. Similar results with CD4+ T-cell depletion and reduced therapeutic response were observed when a group used a stimulator of interferon genes (STING) agonist alongside OX40R agonist with PD-L1 blockade in a NT2.5 breast cancer model [49]. Using a different strategy, another group showed that combination therapy of autologous whole cell vaccination with anti-PD-1 increased Th1 bias [42], while the aforementioned TWIST1-sPD-1/anti-CTLA-4 study also showed increased Th1-like CD4+ T-cells and decreased Th2-like CD3+ cells [43]. However, some studies with checkpoint blockade vaccine strategies showed CD4+ T-cell depletion had little bearing on CD8+ T-cell function [44,45]. Future studies will need to further characterize CD4 and CD8 T-cell phenotypes to determine their activity and function in their response to BC vaccines and checkpoint blockade adjuvants.

### 3.2. Tregs

CD4+ CD25+ FoxP3+ Treg cells are a major immunosuppressive cell and, while they prevent deleterious autoimmune diseases [50], their presence in the breast cancer TME are a significant hurdle for effective BC vaccine responses [51,52]. Tregs directly suppress leukocytes of the TME and secrete immunosuppressive cytokines such as IL-10. HER2 peptide nelipepimut-S (E75) vaccination strategies for patients with HER2+ cancer may aid in reducing the amount of circulating Tregs [53], highlighting the potential of BC vaccines to target this immune cell type. Presently, two vaccine adjuvant strategies are being evaluated in clinic to block Treg cells are adjuvant cyclophosphamide [54] (CP; NCT03066947) or Treg blocking antibodies (NCT01660529).

CP is a commonly used alkylating chemotherapy for patients with BC and is currently being evaluated as adjuvant for BC vaccines. Given at routine low doses, CP significantly impacts Tregs and endothelial cells (antiangiogenic), perhaps by downregulating the TGF-β receptor [55] or via ATP-dependent proliferation-dependent cytotoxicity [54]. In patients with advanced breast cancer, metronomic CP administration transiently decreases Tregs while increasing effector CTLs [56]. In a clinical study employing twenty patients with HER2+ BC, an allogenic GM-CSF secreting vaccine with CP adjuvant increased disease free survival at both seven-month and 42-month timepoints [57]. In this study, Tregs were depleted across all vaccination cycles, including Tregs with CTLA-4 expression. In addition to this study, additional clinical and preclinical studies have tested adjuvant CP for oncolytic virus therapy, folate receptor peptide vaccines, and other vaccine approaches [58,59,60,61,62].

Two other strategies to suppress TME Tregs in patients with breast cancer are anti-FoxP3 [63] anti-CD25 treatment [64]. Anti-FoxP3 treatment with a neutralizing peptide (p60) in a 4T1 model with DC-based vaccines improved survival outcomes and decreased lung metastasis more than p60 or vaccine alone [63]. This treatment works via suppressing IL-10 and reducing TME immunosuppression. The other strategy, anti-CD25 treatment (daclizumab), has been interrogated in a clinical trial including patients with metastatic BC receiving an hTERT/survivin based peptide vaccine. The treatment was well tolerated, with statistically significant decline of FoxP3+ CD4 Treg cells at multiple time points. At the two-year point, the majority of patients in the study living with mBC disease were still alive [64]. Ultimately, CP, anti-FoxP3, and anti-CD25 need to be further explored in human clinical trials as vaccine adjuvants.

## 4. Myeloid Cells

Dendritic cells (DCs) of the TME, sometimes called “natures adjuvants”, are crucial in activating CD8+ and CD4+ T-cells by presenting antigens via MHC I and MHC II, respectively [65]. Moreover, they are important costimulatory cells for T-cell activation because they secrete IL-12 and express CD80/86 to bind CD28 on T-cells. However, DCs in the TME are often immature and, instead of activating T-cells, they may suppress them while also promoting tumor growth and angiogenesis [66,67]. In cancer models, immature DCs are responsible for facilitating Th2 CD4+ phenotypes, which may facilitate tumor growth [68]. Other immunosuppressive cells in the TME, such as M2 TAMs and MDSCs, as well as signals from tumor cells themselves, such as vascular endothelial growth factor (VEGF), CCL2, and IL-10, are involved in restricting DC maturation. Moreover, CTLA-4 expressed by breast cancer cells may attenuate CD80/86 DC expression and subsequent restricted maturity can hinder proper CD8+ and Th1 CD4+ effector cells [69]. In patients, heavy infiltration of primary breast tumor with CD207+Langerin+ immature DCs are a common pathological feature and, while their presence does not directly affect prognosis in themselves, it is likely they are blunting the immune response to immunotherapies [70,71]. Understanding DC biology in the TME has empowered researchers to modulate DCs in the TME to employ effective vaccine strategies.

There are two ways to mobilize dendritic cells in BC vaccine strategy. First, in the direct DC vaccine strategy, autologous myeloid-lineage cells can be pulsed ex vivo to increase specific antigen presenting capabilities and then reinfused into the patient. Secondly, adjuvants that stimulate DCs in vivo can be used to stimulate maturity, such GM-CSF (granulocyte-macrophage colony-stimulating factor), CD40 agonists, TLR agonists, or lentiviral administration of antigens [72] with or without the use of DC vaccines. Thus, both ex vivo and in vivo strategies are sometimes used in tandem.

### 4.1. DC Vaccines

The goal of effective DC vaccine strategies is to create a proinflammatory TME with effective tumor-specific antigen presentation characteristic of mature DCs. In this process, naïve mononuclear cells or immature DC cells are collected from patients and “pulsed” with TSA/TAA antigens [73,74] or autologous cancer cells [75] to prime activation and antigen presentation before re-injection as a vaccine. Sometimes, the DCs are fused to BC cells in vaccine production [76,77]. Moreover, adjuvants such as IL-12, IFN-γ, lipopolysaccharides (LPS), and other cocktails are used to induce a mature DC phenotype [78,79,80,81,82]. These pulsed and primed DCs are then inoculated back into the patient or animal model as a vaccine. Recent discoveries have improved the effectiveness of DCs as pro-inflammatory APCs, thereby creating an effective pro-inflammatory TME response. For instance, recent experiments show that microRNAs can further improve maturation and efficacy of dendritic based vaccines, either employing them directly on DCs or tumor-derived exosomes (TEX) interacting with DCs [83,84,85]. Another effective strategy employs Th1 cytokines, which can increase the efficiency of DC vaccines [86,87]. This rationale is based on successful DC vaccine or DC-targeting vaccine strategies that not only activate effector CD8+ T-cells, but also elicit a Th1 CD4+ proinflammatory response [88,89].

In a clinical trial with patients with ER-PR-double negative BC, a DC vaccine was primed with autologous tumor cells before four intradermal injections with no significant reported adverse events [90]. While overall survival was not affected comparing vaccinated and non-vaccinated participants, the progression free survival over three years was significantly improved in patients receiving the DC vaccine. The vaccinated patients had Th-1 mediated response in addition to increased peripheral CD8+ T-cells and NK cells. Currently, there are DC vaccine strategies employed for clinical trials incorporating priming from HER2, MUC1, autologous cancer cells, and personalized neoantigens, many of which have proven to be safe and effective (see Table 2) [81,82,91,92,93].

### 4.2. DC-Targeting In Situ Vaccination

In vivo modulation of TME (referred sometimes as in situ vaccination [13]) affects anti-tumor utility of DCs with or without use of ex vivo vaccination strategies. Combining tumor-DC fusion vaccines with IL-12, an in situ DC stimulator, increases vaccine effectiveness in preclinical trials using MCF-7 BC cells [94] and has been tested in a clinical trial involving BC patients (NCT00622401). However, this trial was terminated due to vaccine related toxicities. In a novel in situ strategy, another group of investigators achieved robust anti-tumor response in the 4T1 breast cancer model by pulsing dendritic cells with tumor-cell derived exosomes that were fused with the DAMP, nucleosome-binding protein 1 (HMGN1; TEX-N1ND) [95]. This strategy works by exposing DCs to multiple tumor antigens while simultaneously exposing DCs to signs of infection. With TEX-N1ND treatment, tumor-infiltrating CD8+ CTLs and peripheral blood CD8+ T-cells significantly expanded. In addition, there were marked increases in costimulatory molecules (CD83/86; MHC I/II) along with IFN-γ and IL-2 cytokines. These novel approaches present new paradigms in which to test existing cancer vaccines.

In similar fashion to DAMP-rich exosomes, TLR agonists can be used alongside DC vaccines to activate pro-inflammatory TME by introducing a PAMP (see NCT03789097). TLR8 agonism increases IL-12 and TNFα secretion via DCs using the small molecule, VTX-2337, while also sensitizing patient BC samples to Rituximab-dependent-cell mediated toxicity [96]. Poly-ICLC (Hiltonol) is a TLR3 agonist comprised of synthetic dsRNA, which stimulates the production of pro-inflammatory cytokines and IFN-γ [97,98,99,100]; however, there are mixed results regarding TLR3’s role in tumor cell intrinsic phenotypes [101,102]. Poly-ICLC is currently being used as an adjuvant to an in-situ vaccine strategy for advanced BC that also includes pembrolizumab, radiation, and FMS-like tyrosine kinase 3 ligand (Flt3L), which primes DCs for maturation and antigen recognition (NCT03789097). Flt3L binds a receptor tyrosine kinase in the TME. This study being led by Brody and colleagues has shown that this vaccine strategy induces successful antigen cross-presentation in TLR3+ DCs of tumor microenvironment and subsequent tumor regression in a clinical trial of indolent non-Hodgkin’s lymphoma [103]. Upon activation, mature DCs expand and secret proinflammatory cytokines. This work is congruent a with recent study showing E0771 BC cells respond well to adoptive T-cell therapy, with T-cells expressing Ftl3L, combined with Poly-ICLC [104]. Future and ongoing trials will evaluate the translational potential of these in situ vaccination approaches targeting DC cells with and without the use of ex vivo pulsed DC vaccines.

GM-CSF, while having multiple effects across different immune cell types, is a growth factor with strong positive influences on DC recruitment and maturation. It has been used safely by itself or as a BC vaccine adjuvant in many clinical trials, with varying success [57,105,106,107,108,109,110,111]. In fact, combination of GM-CSF with Flt3L may further increase peripheral DC counts in patients [112]. In one study, a triple therapy of GM-CSF autologous BC vaccine, trastuzumab, and cyclophosphamide for HER2+ BC conferred a 6-month clinical benefit rate of 55% [57], which was hypothesized to work via a DC-driven augmentation of CD8+ CTLs [113]. Using a metastatic BC cell line overexpressing GM-CSF (SV-BR-1-GM), BriaCell Therapeutics demonstrated that these cell lines can present not only TAAs, but also MHC I/II molecules and directly act as APCs [114]. In a preclinical study with a Balb/c 4T1 orthoptic BC model, a GM-CSF-based vaccine strategy significantly decreases tumor growth and increases overall survival [115]. Currently, GM-CSF, administered or overexpressed transgenically within tumor, is being evaluated as an adjuvant in clinical trials with HER2 peptide BC/DCIS vaccines (NCT00791037, NCT02636582, NCT02276300, NCT02297698, NCT00524277), folate receptor alpha peptide BC vaccines (NCT02593227, NCT03012100), folate binding peptide BC vaccines (NCT02019524), autologous BC vaccines (NCT00317603, NCT00880464), estrogen receptor peptide BC vaccines (NCT04270149), DNA-based BC vaccines (NCT02790401, NCT00436254), and others.

Immune checkpoint blockers may modulate the TME via DC-driven mechanisms as part of another in situ-based vaccination strategy. DC vaccines in combination with checkpoint inhibitors result in robust immune responses [46,116]. Pembrolizumab is currently being tested as an adjuvant in ongoing DC vaccine clinical trial in the setting of TNBC brain metastasis (NCT04348747). In addition to PD-1/PD-L1 inhibition, other checkpoint inhibition strategies appear to work via DCs and may offer novel avenues of DC vaccine adjuvants. Checkpoint inhibition with an anti-T cell Immunoglobulin and Mucin Domain-containing Protein 3 (TIM3) antibody, for instance, suppresses tumor growth via CD103+ DCs. When this antibody was administered alongside nab-paclitaxel in a *MMTV-PyMT* BC model, dendritic cells activated CD8+ CTL responses via CXCL9 [117]. Therefore, checkpoints inhibitors along with DC activation via vaccines may be an attractive therapeutic route.

Lastly, already established BC treatments also rely heavily on DCs. Immunogenic doses of radiation targeting breast cancer cells, for instance, release breast cancer dsDNA in exosomes, which cause STING-mediated IFN-type-1 activation in DCs of TME [118]. Interestingly, in addition to nurturing mature DCs, radiation therapy may also increase endothelial activation, which recruits leukocytes via upregulation of adhesion proteins [119]. Radiation, poly-ICLC, and DC vaccine triple therapy has been tested in a phase I trial for advance cancer, including BC, and appears to be safe and immunostimulatory [120]. In addition to radiation therapy, other common BC therapies such as trastuzumab increase soluble HER2/neu uptake and presentation by DCs in HER2+ models of BC [121]. Meanwhile, nab-paclitaxel in combination with DC injection increase anti-tumor responses in a DA3 cancer model [122]. While these established therapies have been traditionally viewed as immunosuppressive because they are cytotoxic, the recent literature has opened up new avenues of TME exploitation that mat benefit immunotherapy efficacy. These preclinical studies that interrogate the microenvironment may help guide future clinical trial design with DC vaccines or other BC vaccine strategies.

### 4.3. MDSCs

Myeloid derived suppressor cells (MDSCs), another major component in the TME, suppress TILs, promote tumor angiogenesis, and support tumor growth [123,124]. For instance, CXL2/CCL2 from TNBC cells recruit MDSCs, which then promote stem cell phenotypes and metastatic behavior in TNBCs via chitinase-3-like protein-1 and matrix metallopeptidase-9 [125].

MDSC targeting adjuvants could potentially be used as vaccine adjuvants. For example, poly-ICLC (TLR3 agonist; vaccine adjuvant discussed *vide supra*) decreases tumor and peripheral MDSCs [126]. The presence of MDSCs can also be reduced with entinostat (a HDAC inhibitor) in conjunction with checkpoint inhibitors [127]. Doxorubicin pretreatment, followed by FAP/survivin DNA vaccine, reduces 4T1 TNBC cell growth by eliminating MDSCs [128]. These observations are consistent with other in situ, B-cell based, and other vaccine strategies that eliminate MDSCS [129,130,131]. Additional work is required to interrogate the role of MDSCs in vaccine success and test MDSC-targeting adjuvants in BC vaccine clinical trials.

## 5. Oncolytic Viruses

Oncolytic viruses (OV) transform immunotherapy from a sterile immunity to a pathogen-associated-like immunity, as these viruses replicate preferentially in tumor cells and release TAAs and PAMPS [132,133]. In this emerging in situ vaccination strategy in breast cancer models, pox- [134,135], reo- [136], herpes simplex [137,138,139], adeno- [140,141,142,143], Newcastle disease [144,145], Vesicular stomatitis [146], and measles [147,148,149] viruses have been employed to make breast tumors more immunogenic and more responsive to immunotherapies by priming the TME to signs of infection and releasing TAAs [150]. Moreover, these viruses can be engineered to express various TAAs to further prime an immunological response from TME. In the last couple years, the field of oncolytic viruses in BC models has been burgeoning and opened new avenues for combination immunotherapies, such as vaccines [146,151], suppressor cell targeting [152], and checkpoint inhibitors [134,140,146,150,152,153,154,155,156]. Moreover, several BC clinical trials are currently ongoing (see Table 2).

### 5.1. In Situ TME Changes with Oncolytic Viruses

OVs have been appreciated for many years for their preferential affinity for malignant-cells [148], but the recent explosion in immunotherapies, virology, and molecular genetics have truly opened up new potential to combine OVs with checkpoint inhibitors, adoptive T transfer, and BC vaccines [132]. In 2015, the Food and Drug Administration (FDA) approved the first OV therapy—a GM-CSF-based HSV1—for unresectable melanoma [157]. Researchers have leveraged the cancer cell’s compromised innate immune system, such as faulty TLRs, IFNs, and protein kinase- R (PKR) pathways, which allows a viral infection in the TME that spares normal cells [132]. New technologies have primed OVs to become more specific and effective in targeting tumor cells and lysing them in the process, including arming them with pro-apoptotic signaling molecules [147]. Importantly, the lysing of tumor cells releases TAAs and DAMPs from the cancer cells and PAMPs from the virus particles. Recently, numerous periclinal BC experiments have demonstrated promising results with clinical trial candidates, many of which have entered phase I/II clinical trials [133,143] (see Table 2).

There are currently many creative applications of OVs in recent years, which show very promising results in preclinical BC models. Some of the strategies in breast cancer engineer the viruses to express immune stimulatory molecules or cytokines, such as IL-2 [152], IL-12 [137,144], and GM-CSF [153]. In other instances, researchers have engineered viral-like nanoparticles with immune stimulatory molecules such as IL-33 [158]. Recently, a group of investigators engineered an oncolytic adenovirus expressing a TGF-β decoy (sTGFβRIIFc), which inhibited pro-tumorigenic signaling from fibroblasts in the TME of TNBC models [140]. This strategy was combined with checkpoint inhibitors in a 4T1 Balb/c model, which led to significant tumor regression. In a similar strategy, Zhao et al. expressed decorin, a TGF-β inhibitor, in an oncolytic adenovirus, which significantly reduced lung metastasis in a 4T1 Balb/c model when injected intravenously [142]. While interrogating a mechanism of decreased lung metastasis, they demonstrated CD8+ T-cell augmentation and a CD4+ Th1 bias in the lung with the presence of IL-2, IL-12, and TNFα, with a corresponding decrease in Th2 cytokines and angiogenic signals such as VEGF. In yet another strategy, an interferon small molecule inhibitor led to increased intratumor oncolytic herpes simplex replication, which increased the infiltration of M1 polarized macrophages in the TME of a TNBC xenograft model. Other research groups have expressed CD40ligands in adeno-OV with similarly successful results [159]. Therefore, the in-situ changes with the use of OVs primes the TME for inflammation, opening the door to enhance antigen presentation and attack via CD8 T-cell-inducing vaccines [132].

### 5.2. Oncolytic Viruses Combined with Peptide Vaccines

Niavarani, et al. have recently employed an autologous 4T1 cell vaccine (inactivated by gamma-irradiation) infected with Recombinant Vesicular Stomatitis Virus (VSVd51) [146]. The vaccine was injected subcutaneously in TNBC models using MDA-MB-231, BT-549, and 4T1 TNBC cell lines. VSVd51 is a rhabdovirus engineered to preferentially infect cancer cells via a point mutation in gene encoding its matrix M protein. Cancer cells are preferentially targeted by VSVd51 because they are unable to mount a proper IFN response, whereas normal cells can. Upon administration of VSVd51, the tumors become necrotic with intratumoral expression of pro-inflammatory gene signatures including MHC I, CCL5, and CXCL10 in all three TNBC models. In the 4T1 model, IFN-γ, IL-2, and PD-1 were also upregulated. In the 4T1 model, there was marked increase of multiple NK cell subtypes, CD11c+ CD86+ DCs, and effector CD8+ CTLs in infection-vaccine combination compared to either VSVd51 or vaccine alone. In fact, anti-tumor results in the combination treatment group were dependent on CD8+ T-cell infiltration as their removal abrogated the vaccine-OV response.

Of interest, based on observation of CD8+ T-cell infiltration, Niavarani et al. combined their vaccine-OV approach with anti-PD-1 treatment, which improved overall survival compared to PD-1 or vaccine-OV alone in a 4T1-Balb/c model [146]. This might be an indication of a wider trend in BC-OV research, as multiple groups have seen BC tumor regression and increased survival by combining OVs with checkpoint inhibitors [134,140,146,150,152,153,154,155,156]. Chon et al., for instance, used antibodies blocking both CTLA-4 and PD-1 in a *MMTV-PyMT* transgenic mouse model administration of mJX-594 (JX), a vaccinia virus engineered to express GM-CSF and is attenuated via viral thymidine kinase disruption [153]. JX combined with anti-CTLA-4 and anti-PD-1 significantly decreased tumor burden and lung metastasis while increasing overall survival compared to any other group. Remarkably, the triple therapy impacted the TME to a pro-inflammatory state, with evident increase of CD8+ T-cell infiltration. In the same study, the investigators saw decreased CD31+ (tumor angiogenesis), with marked increase of CD8+ cytotoxic T-cells, CD11c+ DCs, and PD-L1+ in the TME of a Renca Balb/c model, showcasing even more drastic TME changes than a *MMTV-PyMT* orthotopic model. Future studies can continue to interrogate TME immune and vascular cells, while testing OV, vaccine, and checkpoint inhibitor combination therapy. In fact, a reo-OV (Pelareorep) is currently being tested in combination with anti-PD-L1 (avelumab) and nab-paclitaxel for patients with ER+HER2- metastatic breast cancer (NCT04215146).

## 6. Microenvironment Antigen Vaccines

### 6.1. Tumor Endothelia

Vascular cells in the TME affect tumors in multiple ways: (1) blood supply to the tumor, (2) infiltration of CTLs via endothelial adhesion proteins, similar to inflammatory processes [21,160], and (3) the intravasation and extravasation of circulating tumor cells (CTC) during the metastatic process, similar to the leukocyte extravasation pathways [161,162]. Pathological angiogenesis in the vascular TME can suppress effective immunotherapies, which can potentially be overcome by antiangiogenesis strategies that “normalize” the endothelium [163]. Antiangiogenic anti-VEGF therapy targeting via bevacizumab (Avastin) lost its indication status for BC in 2011 from the FDA, but its use remains controversial as it is relatively safe and confers minor benefits for select patients [164]. Anti-VEGF therapy is prone to resistance and adverse events (due to hypoxia-induced pro-metastatic phenotypes) across many cancers [165]. Researchers have resorted to alternative angiogenesis targeting methods in preclinical studies. For instance, targeting Notch ligands with decoys that utilize the EGF-like repeats of the Notch1 receptor decrease tumor growth and angiogenesis in orthotopic breast cancer models with minimal adverse events [166]. Moreover, angiogenesis is an emerging target for BC vaccine strategies, mostly in the preclinical phase. In the clinic, a recently completed phase I DC vaccine strategy pulsed DC cells with tumor blood vessel antigens (TBVA) (NCT02479230).

Yu-Quan Wei was the first to pioneer “endothelial cell vaccines” around twenty years ago [167] and, since then, multiple strategies have been tested in preclinical models of BC [168]. There are several endothelial vaccine strategies that have been tested, including whole-cell endothelial-based [169,170], TBVA-targeting [171], EGFR-targeting [172,173], CD105-targeting [174,175], PDGFR- β -targeting [176], and VEGF targeting [169,173,177,178,179,180] vaccines. Not only do the vaccines attack tumor growth by directing immune responses against tumor angiogenesis, but the inflammatory consequences of the immune attack lead to increased CD8+ CTLs infiltrating into the TME [167,170]. In one of the studies, a mutant VEGF (VEGF165b) was used alongside MUC1 in a peptide vaccine strategy in a EMT-6 Balb/c BC model [180]. The strategy increased antibody titers to wildtype VEGF 200,000-fold while reducing Tregs and increasing MUC1-specific CD8 T-cells. Moreover, the authors showed an ability of vaccine mediated anti-VEGF antibodies taken from serum to inhibit endothelial cells from proliferating in vitro. This study is an example in which both tumor antigen presentation and pro-inflammatory TME elements are increased by combining TAA and TME antigen vaccination strategies.

Recent transcriptional analysis of the endothelium in homeostasis and disease states has revealed striking heterogeneity depending on vascular bed or disease context [181,182]. Further understanding of the tumor vasculature may increase our ability to target tumor endothelial antigens in vaccination strategies with or without TME-stimulating adjuvants.

### 6.2. CAFs

Cancer associated fibroblasts (CAFs) of the TME are transcriptionally, genetically, and epigenetically unique compared to the surrounding fibroblasts of normal tissue, with notable upregulation of Notch2 and genome-wide methylation signatures [183,184,185]. CAFs promote pro-tumorigenic and metastatic phenotypes, as demonstrated by xenograft modelling [186] and single cell analysis of immune-resistant cancer cell populations [184].

There are two main CAF-vaccine strategies. First, CAF-associated antigens have been targeted by DNA and recombinant peptide vaccines [128,187,188,189,190]. Two of these vaccines, for instance, targeted fibroblast activation protein-alpha (FAP-α) using DNA-based vaccines in 4T1 mouse models [187,188,189]. Targeting of FAPa led to a decrease in CAFs present, which resulted in decreased CCL2/CXCL12 expression and MDSC presence. This resulted in increased anti-tumor response and overall survival. The other CAF-related strategy employs allogenic fibroblasts transfected to express TAAs to promote immune response [191,192]. Essentially, immune-stimulatory CAFs are engineered to present tumor antigens and initiate a pro-inflammatory anti-tumorigenic environment. It has yet to be seen whether these preclinical studies will be translated in a meaningful way to help patients.

## 7. Summary and Future Directions

The immune system has evolved over the course of millions of years primarily in an arms race against pathogens. Anti-cancer immunity, however, poses a more delicate balance as the immune system must navigate a complicated terrain of “self” versus “non-self” in which autoimmune pathology is at odds with cancer immunosurveillance. At the heart of this balance is the TME, which is instrumental not only in supporting an oncogenic niche, but also in deciding whether or not the immune cells of the local environment will become active once they recognize immunogenic properties of the tumor. Therefore, while BC vaccines can help with antigen presentation, the rate limiting step may be outside the realm of TSAs and TAAs. Instead, it may be within the TME. Multiple TME-targeting vaccine-based clinical trials (see Table 2) are underway for patients with various BC subtypes. While checkpoint inhibitors for cases of TNBC are leading the forefront of BC immunotherapies, BC vaccine strategies are now also targeting or utilizing immunostimulatory molecules, immunosuppressive cells, and other components of the TME in order to develop effective therapeutic BC vaccines as part of combination strategies.

## Figures and Tables

**Table 1:** BC Vaccine Types.

Class	Examples
TSA Peptide Vaccines	Cancer-testis antigens, Neoantigens, Tumor virus antigens
TAA Peptide Vaccines	HER2/neu (eg E75, NeuVax), MUC1, p53, CEA, hTERT, Folate Binding Proteins (E39/J65), sialyl Lewisª
DNA Vaccines	Rat HER2/neu, Human mammaglobin-A (SCGB2A2), CD105/Yb-1/SOX2/CDH3/MDM2-polyepitpope plasmid, neoantigen DNA, pUMVC3-IGFBP2-HER2-IGF1R plasmid, pNGVL3-hICD plasmid
RNA Vaccines	IVAC_W_bre1_uID and IVAC_M_uID (IVAC MUTANOME), Alphavirus-like replicon particles with HER2 RNA
Whole Cell Vaccines	Dendritic Cell (DC) vaccines, Autologous or allogenic tumor vaccines
Microenvironment Targeting Vaccines	Angiogenesis-targeting vaccines, Fibroblast-targeting vaccines

**Table 2:** BC Vaccine Clinical Trials that Target TME.

Clinical Trial ID	BC Type	Vaccine Description	Microenvironment Target	Phase
*NCT03362060*	mTNBC (HLA-A2+)	PVX-410 (Multi-peptide Vaccine) + Pembrolizumab (Anti-PD-1)	Checkpoint Molecules	I (recruiting)
*NCT03066947*	Local or mBC	SV-BR-1-GM (GM-CSF Secreting BC Cell Line) + Cyclophosphamide	Dendritic Cells and Other Cells (GM-CSF); Tregs	I/II (completed)
*NCT02479230*	BC	Type I Polarized Autologous DC Vaccine with Tumor Blood Vessel Antigen-Derived Peptides	Dendritic Cells; Angiogenesis	I (completed)
*NCT01730118*	HER2+ BC	HER2-pulsed DC Vaccination	Dendritic Cells	I (completed)
*NCT02018458*	TNBC	Cyclin B1/WT-1/CEF-loaded DC Vaccine + Chemotherapy (Varied)	Dendritic Cells	III (completed)
*NCT02063724*	HER2+ BC	HER2-pulsed DC Vaccine	Dendritic Cells	I (active, not recruiting)
*NCT02061423*	HER2+ BC	HER2-pulsed DC Vaccine + Trastuzumab	Dendritic Cells	I (active, not recruiting)
*NCT02061332*	DCIS	HER2-pulsed DC Vaccine	Dendritic Cells	I/II (completed)
*NCT02643303*	BC	Poly-ICLC in situ Vaccine + Durvalumab (Anti-PD-1) + Tremelimumab (Anti-CTLA-4)	TLR; Checkpoint Molecules	I/II (recruiting)
*NCT00791037*	HER2+ BC	HER2 Peptide Vaccine + Aargramostim + Cyclophosphamide + Adoptive HER2 Specific T-cells	Dendritic Cells and Other Cells (GM-CSF); T-cells; Tregs	I/II (completed)
*NCT02140996*	BC	Ad-sig-hMUC1/ecdCD40L Vector Vaccine	APCs (CD40L)	I (recruiting)
*NCT02593227*	BC	Folate Receptor Alpha Peptide Vaccine + GM-CSF + Cyclophosphamide	Dendritic Cells and Other Cells (GM-CSF); Tregs	II (active, not recruiting)
*NCT02636582*	DCIS	NeuVax (Nelipepimut-S Peptide Vaccine with Sargramostim Adjuvant)	Dendritic Cells and Other Cells (GM-CSF)	II (active, not recruiting)
*NCT01660529*	mBC	hTERT/survivin/CMT Multipeptide Vaccine + Basiliximab (anti-CD25)	Tregs	I (completed)
*NCT01570036*	HER2 low BC	NeuVax (Nelipepimut-S Peptide Vaccine with Sargramostim Adjuvant) + Trastuzumab	Dendritic Cells and Other Cells (GM-CSF)	II (completed)
*NCT04215146*	ER+ HER2- mBC	Pelareorep (Reovirus-based Therapy) + Nab-paclitaxel + Avelumab (Anti-PD-L1)	Multi-target (Oncolytic Virus); Checkpoint Molecules	II (recruiting)
*NCT02779855*	TNBC	Talimogene Laherparepvec (Herpes Virus-based Therapy) + Nab-paclitaxel	Multi-target (Oncolytic Virus)	I/II (active, not recruiting)
*NCT04301011*	TNBC	TBio-6517 (Vaccinia Virus-based Therapy) + Pembrolizumab (Anti-PD-1)	Multi-target (Oncolytic Virus); Checkpoint Molecules	I/II (recruiting)
*NCT03740256*	HER2+ BC	CAdVEC (Adenovirus-based Therapy) + Autologous CAR Viral Specific T-cells	Multi-target (Oncolytic Virus); T-cells	I (not yet recruiting)
*NCT02826434*	TNBC	PVX-410 (Multi-peptide Vaccine) + Durvalumab (Anti-PD-L1)	Checkpoint Molecules	I (active, not recruiting)
*NCT02276300*	HER2+ BC	HER2 Peptide Vaccine + Sargramostim + Cyclophosphamide + Imiquimod	Dendritic Cells and Other Cells (GM-CSF); Tregs, TLR	I (completed)
*NCT02297698*	HER2+ BC	NeuVax (Nelipepimut-S Peptide Vaccine with Sargramostim Adjuvant) + Trastuzumab	Dendritic Cells and Other Cells (GM-CSF)	II (active, not recruiting)
*NCT00986609*	TNBC	MUC-1 Peptide Vaccine + Poly-ICLC	TLR	I (completed)
*NCT02019524*	BC	Folate Binding (E39 and J65) Peptide Vaccine + Sargramostim	Dendritic Cells and Other Cells (GM-CSF)	I/II (completed)
*NCT00524277*	HER2+ BC	HER2 (GP2) Peptide Vaccine + Modified HER2 (AE37) Peptide Vaccine + Sargramostim	Dendritic Cells and Other Cells (GM-CSF)	II (completed)
*NCT00971737*	BC	Allogenic GM-CSF-Secreting Whole Tumor Cell Vaccine + Cyclophosphamide	Dendritic Cells and Other Cells (GM-CSF); Tregs	II (completed)
*NCT03066947*	BC	SV-BR-1-GM (GM-CSF Secreting BC Cell Line) + Cyclophosphamide + IFN-α-2b	Multiple Targets (Interferon); Tregs	I/II (completed)
*NCT00622401*	BC	DC-Tumor Fusion Vaccine + IL-12	Dendritic Cells; Other (IL-12)	I/II (terminated)
*NCT00317603*	mBC	Autologous GM-CSF-Secreting BC Cell Vaccine	Dendritic Cells and Other Cells (GM-CSF)	I (active, not recruiting)
*NCT02427581*	TNBC	Personalized Synthetic Long Peptide Breast Cancer + Poly-ICLC	TLR	I (recruiting)
*NCT04024800*	TNBC	Modified HER2 (AE37) Peptide Vaccine + Pembrolizumab (Anti-PD-1)	Checkpoint Molecules	II (recruiting)
*NCT00880464*	BC	Autologous GM-CSF-Secreting BC Cell Vaccine	Dendritic Cells and Other Cells (GM-CSF)	I (active, not recruiting)
*NCT03199040*	TNBC	Neo-Antigen DNA vaccine + Durvalumab (Anti-PD-1)	Checkpoint Molecules	I (recruiting)
*NCT03632941*	HER2+ BC	Neutralized Viral Vector Vaccine (VRP-HER2) + Pembrolizumab (Anti-PD-1)	Checkpoint Molecules	II (recruiting)
*NCT03384914*	HER2+ BC	DC1 Vaccine + pUMVC3-IGFBP2-HER2-IGF1R (WOKVAC) DNA Vaccine	Dendritic Cells	II (recruiting)
*NCT04270149*	ER+ BC	ESR1 Peptide Vaccine + Montanide + GM-CSF	Dendritic Cells and Other Cells (GM-CSF)	I (not yet recruiting)
*NCT03387553*	HER2+	HER2-pulsed DC Vaccine	Dendritic Cells	I (recruiting)
*NCT04348747*	mTNBC	Anti-HER2/HER3 DC Vaccine + Celecoxib + Pembrolizumab (Anti-PD-1) + IFN-α-2b	Dendritic Cells; Multiple Targets (Interferon); TLR; Checkpoint Molecules	II (not yet recruiting)
*NCT02780401*	HER2- BC	pUMVC3-IGFBP2-HER2-IGF1R (WOKVAC) DNA Vaccine + Sargramostim	Dendritic Cells and Other Cells (GM-CSF)	I (active, not recruiting)
*NCT04105582*	TNBC	Neo-Antigen Pulsed DC Vaccine	Dendritic Cells	I (recruiting)
*NCT03387085*	TNBC	Adenoviral and Yeast-based Vaccines (CEA, Brachyury, MUC1, Mutant RAS) + Bevacizumab (Anti-VEGF) + Avelumab (Anti-PD-L1) + N-803 (IL-15 Agonist) + NK-92 (hNK Cells) + Chemotherapy (Varied) + Radiation	Multi-target (IL-15 agonist); Angiogenesis; Checkpoint Molecules; NK Cells	I/II (active, not recruiting)
*NCT03606967*	TNBC	Personalized Synthetic Long Peptide Vaccine + Nab-paclitaxel + Durvalumab (Anti-PD-1) + Poly-ICLC	Checkpoint Molecules; TLR	II (not yet recruiting)
*NCT03012100*	TNBC	Folate Receptor Alpha Peptide Vaccine. + Sargramostim + Cyclophosphamide	Dendritic Cells and Other Cells (GM-CSF); Tregs	II (recruiting)
*NCT03804944*	ER+ BC	Flt3L + Radiation Therapy + Pembrolizumab (Anti-PD-1)	Multi-target (radiation); Checkpoint Molecule; Dendritic Cells	II (active, not recruiting)
*NCT04197687*	HER2+ BC	HER2 Peptide Vaccine (TPIV100) + Sargramostim + Pertuzumab + Trastuzumab	Dendritic Cells and other cells (GM-CSF)	II (recruiting)
*NCT04418219*	BC	SV-BR-1-GM (GM-CSF Secreting BC Cell Line) + Cyclophosphamide + IFN-α-2b + Pembrolizumab (Anti-PD-1)	Dendritic Cells and Other Cells (GM-CSF); Multi-target (Interferon); Checkpoint Molecules	I/II (not yet recruiting)
*NCT04296942*	mBC	Brachyury-TRICOM (Vaccinia Viral Vector Based Brachyury Vaccine) + Entinostat + Adotrastuzumab Emtansine + M7824 (PD-L1/TGF-β Fusion Protein)	Checkpoint Molecules; Myeloid Cells	I (recruiting)
*NCT01782274*	mBC	Proteomic Approach with Allogeneic Haploidentical Hematopoietic Stem Cells (HSCs) + CTLs + DC Vaccine	Multi-target, including Dendritic Cells	II/III (enrolling by invite)
*NCT03789097*	mBC	Flt3L + Radiation Therapy + Pembrolizumab (Anti-PD-1) + Poly-ICLC	Multi-target (radiation); Checkpoint Molecules;, Dendritic Cells; TLR	I/II (recruiting)
*NCT00436254*	HER2+ BC	HER2 DNA Vaccine + Sargramostim	Dendritic Cells and Other Cells (GM-CSF)	I (active, not recruiting)
*NCT04144023*	DCIS	Multi-epitope HER2 Peptide Vaccine + GM-CSF	Dendritic Cells and Other Cells (GM-CSF)	I (recruiting)
*NCT03564782*	mBC	PVSRIPO (Oncolytic Poliovirus)	Multi-target (Oncolytic Virus)	I (recruiting)
*NCT03328026*	mBC	SV-BR-1-GM (GM-CSF Secreting BC Cell Line) + IFN-α-2b + INCMGA00012 (PD-1 Inhibitor) + Cyclophosphamide Interferon Inoculation + Epacadostat (Indoleamine-pyrrole 2,3-dioxygenase-1 Inhibitor)	Dendritic Cells and Other Cells (GM-CSF); Checkpoint Molecules; Multi-target (Interferon); Tregs	I/II (recruiting)
*NCT04246671*	HER2+ BC	TAEK-VAC-HerBy Vaccine + PD-1/PD-L1 Inhibition	Checkpoint Molecules	I/II (not yet recruiting)
*NCT01376505*	HER2+ BC	HER2 Peptide Vaccine + nor-MDP (Muramyldipeptide Derivative—a Bacterial Cell wall Peptidoglycan)	Multi-target (NOD2 agonist)	I (recruiting)
*NCT02491697*	mBC	DC-CIK Vaccine (DC Cells Co-cultured with Cytokine-Induced Killer Cells) + Capecitabine	Dendritic Cells	II (active, not recruiting)
*NCT02432963*	mBC	Modified Vaccinia Virus Ankara Vaccine Expressing p53 + Pembrolizumab (Anti-PD-1)	Checkpoint Molecules	I (active not recruiting)
*NCT03761914*	TNBC	Galinpepimut-S. (Wilms Tumor-1-Targeting Multivalent Heteroclitic Peptide Vaccine) + Pembrolizumab (Anti-PD-1)	Checkpoint Molecules	I/II (recruiting)
*NCT01997190*	mBC	AdV-tk (Adenovirus-mediated Herpes Simplex Virus Thymidine Kinase Gene Therapy) + Valacyclovir	Multi-target (Oncolytic Virus)	I (active not recruiting)
*NCT03289962*	TNBC	RO7198457 (Individualized mRNA Vaccine) + Atezolizumab (Anti-PD-L1)	Checkpoint Molecules	I (recruiting)

Abbreviations: BC, breast cancer; mBC, metastatic breast cancer; TNBC, triple negative breast cancer; mTNBC, metastatic TNBC; VEGF, vascular endothelial growth factor; TGF-β, transforming growth factor beta; PD-1,programmed cell death protein 1; PD-L1, programmed death ligand 1; CTLA-4, cytotoxic T-lymphocyte-associated protein 4; Flt3L, FMS-like tyrosine kinase 3 ligand; GM-CSF, granulocyte-macrophage colony-stimulating factor; NOD2, nucleotide-binding oligomerization domain-containing protein 2; APC, antigen-presenting cell; DCIS, ductal carcinoma in situ.
